# Supplementary material for: Pan-genome analysis identifies intersecting roles for Pseudomonas specialized metabolites in potato pathogen inhibition
Source: eLife. 2021 Nov 18;10:e71900. doi: 10.7554/eLife.71900 (PMC8719888; doi:10.7554/eLife.71900)
Supplement: Supplementary file 2. [file elife-71900-supp2.docx]

**SUPPLEMENTARY FILE 2**

**Supplementary File 2A**. Bacterial strains used in this study.

| Strain | Description | Reference |
| --- | --- | --- |
| SBW25 | *Pseudomonas fluorescens* SBW25 | (Rainey and Bailey, 1996) |
| LMG 2338 | *Pseudomonas* sp. LMG 2338 (NCPPB 387) | (Mortishire-Smith et al., 1991) |
| *E. coli* DH5α (Subcloning Efficiency™ DH5α™) | *E. coli* *endA*1, *hsdR*17(r_K_-m_K_+), *supE*44, *recA*1, *gyrA* (Nal^r^), *relA*1, Δ(*lacIZYA-argF*)U169, *deoR*, Φ80*dlacΔ(lacZ)M15* | Thermo Fisher Scientific |
| Ps616 - Ps734 | 120 environmental *Pseudomonas* strains collected from RG Abrey Farms in Feb 2015 | This study |
| Ps831 - Ps950 | 120 environmental *Pseudomonas* strains collected from RG Abrey Farms in May 2015 | This study |
| IR1-1 - NS6-8 (see Supplementary File 1) | 192 environmental *Pseudomonas* strains collected from RG Abrey Farms in June 2017 | This study |
| Ps682Δvisc | *Pseudomonas* sp. Ps682 with deletion of a NRPS gene in the viscosin-like BGC | This study |
| Ps682::lux | *Pseudomonas* sp. Ps682 expressing *lux* operon | This study |
| Ps682Δvisc::lux | *Pseudomonas* sp. Ps682 Δviscosin expressing *lux* operon | This study |
| Ps619Δten | *Pseudomonas* sp. Ps619 with deletion of a NRPS gene in the tensin-like BGC | This study |
| Ps619Δhcn | *Pseudomonas* sp. Ps619 with deletion of the HCN BGC | This study |
| Ps619ΔtenΔhcn | *Pseudomonas* sp. Ps619 with deletions in tensin and HCN BGCs | This study |
| Ps619::lux | *Pseudomonas* sp. Ps619 expressing *lux* operon | This study |
| Ps619Δten::lux | *Pseudomonas* sp. Ps619Δtensin expressing *lux* operon | This study |
| Ps619Δhcn::lux | *Pseudomonas* sp. Ps619ΔHCN expressing *lux* operon | This study |
| Ps619ΔtenΔhcn::lux | *Pseudomonas* sp. Ps619ΔtensinΔHCN expressing *lux* operon | This study |

**Supplementary File 2B**. Primers used in this study.

| No. | Name | Sequence |
| --- | --- | --- |
| 1 | Viscosin up NdeI | GGAATTCCATATGTCACAGGACGATCTCCATTCGTTC |
| 2 | Viscosin up XbaI | GCTCTAGACCGTGGCCTGCGTATCGAACTG |
| 3 | Viscosin dn XbaI | GCTCTAGACTTGCAGCGGTTCGTCGAG |
| 4 | Viscosin dn BamHI | CGGGATCCGCAGTTCAGCGAATTATTGGCAG |
| 5 | Viscosin ext | GACTTACCAGGCAGGCAGGTTAC |
| 6 | Tensin up Fwd | CGCATATGGGCCGTCATGCCACCACG |
| 7 | Tensin up Rev | CGCCTAGGGTGGCGTCGTTGACCCTG |
| 8 | Tensin dwn Fwd | CGCCTAGGGCAGGATGTCGTGGCGATC |
| 9 | Tensin dwn Rev | CGTCTAGATCAAGTGAATGTGCTCGAACTATTGGCA |
| 10 | Tensin Int Screen Fwd | CCTGACGCCAGACCACTTG |
| 11 | TensinExtr_f | CGTTTGCCCTTCTTGAGTGA |
| 12 | Tens619InterRev | TTGGGAGAACAAGGTCGGTA |
| 13 | Pf_HCN_Up_Fwd | CGGAGCTCCCCCAATCACAGCAACAATAA |
| 14 | PfHCN_UP_Rev | CGTCTAGAGCCATCGAGTACCACCAAA |
| 15 | PfHCN_down_Fwd | CGTCTAGATGAAAGGCCAGATTCTACT |
| 16 | PfHCN_Down_R_Rev | CGCATATGTGTCAGTCAACCTATTCGT |
| 17 | SacB Fwd | GTTGATTGTTTGTCTGCGT |
| 18 | SacB Rev | TTTAGTTCTTTAGGCCCGT |
| 19 | PTS1f | CGGCAGGTATATGTGATGG |
| 20 | PTS1r | GTGAGAAATCACCATGAGTG |

**Supplementary File 2C**. Plasmids used in this study.

| Plasmids | Description | Reference |
| --- | --- | --- |
| pTS1 | pME3087 derivative containing a *sacB* counter-selection marker | (Scott et al., 2017) |
| pTS1-Δviscosin | Construct for viscosin deletion, fragments amplified with primers 1- 2 and 3-4, | This study |
| pTS1-Δtensin | Construct for tensin deletion, fragments amplified with primers 6-7 and 8-9, | This study |
| pTS1-Δ619HCN | Construct for HCN cluster deletion, fragments amplified with primers 13-14 and 15-16 | This study |
| pTNS2 | Tn7 transposase expression plasmid | (K.-H. Choi et al., 2005) |
| pUC18-mini-Tn7T-Gm-lux | mini-Tn7 *luxCDABE* transcriptional fusion vector | (K.-H. Choi et al., 2005) |

**Supplementary File 2D**. NMR data for the isolated viscosin I (600 MHz, DMF-d7, 298 K). Atom numbering and spectra are shown in Figure 5 supplementary figures.

| **Number** | **δ^1^H** | | **δ^13^C** |
| --- | --- | --- | --- |
| **HDA^a^** |  |  |  |
| 1' |  |  | 173.17 |
| 2' | H2 | 2.45 | 44.39 |
| 3' | H | 4.01 | 68.10 |
| 4' | H2 | 1.47 | 38.13 |
| 5' | Ha | 1.46 | 25.92 |
| 5' | Hb | 1.35 |  |
| 6' | H2 | 1.28 | 29.44 |
| 7' | H2 | 1.28 | 29.44 |
| 8' | H2 | 1.27 | 31.94 |
| 9' | H2 | 1.28 | 22.67 |
| 10' | H3 | 0.87 | 13.85 |
| **Leu1** |  |  |  |
| 1NH | H | nd^b^ |  |
| 1a | H | 4.16 | 52.34 |
| 1b | H' | 1.46 | 39.60 |
| 1c | H | 1.73 | 24.84 |
| 1d | H3 | 0.93 | 23.09 |
| 1e | H3 | 0.91 | 21.41 |
| 1CO |  |  | 173.59 |
| **Glu2** |  |  |  |
| 2NH | H | nd^b^ |  |
| 2a | H | 4.16 | 56.87 |
| 2b | H2 | 2.02 | 27.44 |
| 2c | H2 | 2.24 | 34.82 |
| 2d |  |  | 177.25 |
| 2CO |  |  | 173.09 |
| **Thr3** |  |  |  |
| 3NH | H | 8.76 |  |
| 3a | H | 4.38 | 59.73 |
| 3b | H | 5.41 | 70.28 |
| 3c | H3 | 1.34 | 17.37 |
| 3CO |  |  | 173.08 |
| **Val4** |  |  |  |
| 4NH | H | 8.25 |  |
| 4a | H | 3.68 | 63.92 |
| 4b | H | 2.32 | 29.36 |
| 4c | H3 | 1.02 | 20.14 |
| 4d | H3 | 0.95 | 19.15 |
| 4CO |  |  | 173.09 |

^a.^ HDA = 3-hydroxydecanoic acid

^b.^ nd = not determined

| **Number** | **δ^1^H** | | **δ^13^C** |
| --- | --- | --- | --- |
| **Leu5** |  |  |  |
| 5NH | H | 7.08 |  |
| 5a | H | 4.24 | 53.39 |
| 5b | H | 1.88 | 41.34 |
| 5b | H | 1.65 |  |
| 5c | H | 1.75 | 24.72 |
| 5d | H3 | 0.86 | 20.82 |
| 5e | H3 | 0.99 | 22.90 |
| 5CO |  |  | 173.08 |
| **Ser6** |  |  |  |
| 6NH | H | nd^b^ |  |
| 6a | H | 4.26 | 56.86 |
| 6b | H' | 4.07 | 63.10 |
| 6b | H'' | 3.86 |  |
| 6CO |  |  | 171.38 |
| **Leu7** |  |  |  |
| 7NH | H | nd^b^ |  |
| 7a | H | 4.24 | 54.61 |
| 7b | H' | 1.47 | 40.10 |
| 7b | H'' | 1.82 |  |
| 7c | H | 1.75 | 24.84 |
| 7d | H3 | 0.86 | 20.82 |
| 7e | H3 | 0.88 | 22.90 |
| 7CO |  |  | 173.09 |
| **Ser8** |  |  |  |
| 8NH | H | 8.03 |  |
| 8a | H | 4.43 | 56.27 |
| 8b | H' | 3.93 | 61.92 |
| 8b | H'' | 3.74 |  |
| 8CO |  |  | 171.37 |
| **Ile9** |  |  |  |
| 9NH | H | 6.98 |  |
| 9a | H | 4.54 | 56.46 |
| 9b | H | 1.95 | 36.55 |
| 9c | H' | 1.29 | 24.48 |
| 9c | H'' | 1.05 |  |
| 9d | H3 | 0.89 | 11.58 |
| 9e | H3 | 0.86 | 15.52 |
| 9CO |  |  | 169.78 |
