# Supplementary material for: Pan-genome analysis identifies intersecting roles for Pseudomonas specialized metabolites in potato pathogen inhibition
Source: eLife. 2021 Nov 18;10:e71900. doi: 10.7554/eLife.71900 (PMC8719888; doi:10.7554/eLife.71900)
Supplement: Transparent reporting form [file elife-71900-transrepform1.docx]

***eLife’s* transparent reporting form**

We encourage authors to provide detailed information *within their submission* to facilitate the interpretation and replication of experiments. Authors can upload supporting documentation to indicate the use of appropriate reporting guidelines for health-related research (see [EQUATOR Network](http://www.equator-network.org/%20)), life science research (see the [BioSharing Information Resource](https://biosharing.org/)), or the [ARRIVE guidelines](http://www.plosbiology.org/article/info:doi/10.1371/journal.pbio.1000412) for reporting work involving animal research. Where applicable, authors should refer to any relevant reporting standards documents in this form.

If you have any questions, please consult our Journal Policies and/or contact us: [editorial@elifesciences.org](mailto:editorial@elifesciences.org).

**Sample-size estimation**

- You should state whether an appropriate sample size was computed when the study was being designed
- You should state the statistical method of sample size computation and any required assumptions
- If no explicit power analysis was used, you should describe how you decided what sample (replicate) size (number) to use

Please outline where this information can be found within the submission (e.g., sections or figure legends), or explain why this information doesn’t apply to your submission:

Amplicon sequencing sample sizes (Fig. 1) were chosen based on comparison with similar, contemporary experiments. Details are included in the materials and methods section.

The appropriate sample size for whole genome sequencing (Fig. 2) was determined by comparable comparative genomic studies at the onset of this study, as it was difficult to make meaningful assumptions for power analysis. The sample size was similar or exceeded those in the following referenced studies:

PLOS Genet, 2012, 8, e1002784.

PNAS, 2014, 111, E1130.

Environ. Microbiol. 2015, 17, 4764.

**Replicates**

- You should report how often each experiment was performed
- You should include a definition of biological versus technical replication
- The data obtained should be provided and sufficient information should be provided to indicate the number of independent biological and/or technical replicates
- If you encountered any outliers, you should describe how these were handled
- Criteria for exclusion/inclusion of data should be clearly stated
- High-throughput sequence data should be uploaded before submission, with a private link for reviewers provided (these are available from both GEO and ArrayExpress)

Please outline where this information can be found within the submission (e.g., sections or figure legends), or explain why this information doesn’t apply to your submission:

One amplicon sequencing sample showed clear evidence of contamination and as such was excluded from the analyses shown in Figure 1. For completeness, the data was also analyzed including this sample and is shown in Figure 1-figure supplement 1. Source data is also provided for this figure. One sample was removed from the analysis of individual strains as whole genome sequencing determined that this was a mixture of two different *Pseudomonas* strains. Data relating to this sample was therefore not reported or used in any statistical analysis.

All phenotyping (i.e. motility, protease, biocontrol, *S. scabies* inhibition) experiments were conducted at least twice independently for each individual bacterial strain and ordinal values were recorded. For the few isolates where there was a disagreement between the ordinal values from the two experiments, additional repeats were conducted, and a consensus value was assigned. Further details of experimental repeats are recorded in the Materials and Methods.

Genome sequencing data is deposited at the European Nucleotide Archive with the project accession PRJEB34261.

**Statistical reporting**

- Statistical analysis methods should be described and justified
- Raw data should be presented in figures whenever informative to do so (typically when N per group is less than 10)
- For each experiment, you should identify the statistical tests used, exact values of N, definitions of center, methods of multiple test correction, and dispersion and precision measures (e.g., mean, median, SD, SEM, confidence intervals; and, for the major substantive results, a measure of effect size (e.g., Pearson's r, Cohen's d)
- Report exact p-values wherever possible alongside the summary statistics and 95% confidence intervals. These should be reported for all key questions and not only when the p-value is less than 0.05.

Please outline where this information can be found within the submission (e.g., sections or figure legends), or explain why this information doesn’t apply to your submission:

All statistical tests used are described where appropriate in the Materials and Methods, the figure legends and source data files. Numerical data are provided for the data shown in Figure 1 as a source data file. N is defined for data shown in Figure 3 and all Pearson correlation coefficients are shown in Figure 3 – figure supplement 1. N and statistical information for the data presented in Figure 7 is provided in Figure 7 – figure supplement 1, alongside equivalent information for a repeat experiment. N is reported for Figure 8 data. All raw scoring data associated with Figures 2, 3 and 8 are reported in Supplementary File 1.

(For large datasets, or papers with a very large number of statistical tests, you may upload a single table file with tests, Ns, etc., with reference to sections in the manuscript.)

**Group allocation**

- Indicate how samples were allocated into experimental groups (in the case of clinical studies, please specify allocation to treatment method); if randomization was used, please also state if restricted randomization was applied
- Indicate if masking was used during group allocation, data collection and/or data analysis

Please outline where this information can be found within the submission (e.g., sections or figure legends), or explain why this information doesn’t apply to your submission:

No explicit randomization was used. However, following sample collection, strains were assigned anonymous codes based solely on freezer location. Phenotyping was conducted based on these codes by a second team of researchers without reference to the initial sampling sites.

**Additional data files (“source data”)**

- We encourage you to upload relevant additional data files, such as numerical data that are represented as a graph in a figure, or as a summary table
- Where provided, these should be in the most useful format, and they can be uploaded as “Source data” files linked to a main figure or table
- Include model definition files including the full list of parameters used
- Include code used for data analysis (e.g., R, MatLab)
- Avoid stating that data files are “available upon request”

Please indicate the figures or tables for which source data files have been provided:

Source data is linked to Figure 1. Raw numerical data relating to genotyping and phenotyping are included in Supplementary File 1. Hyperlinks to source LC-MS data (as a MassIVE dataset) and associated GNPS analysis are linked to in the Materials and Methods. Parameters for genome assemblies, acquisition of LC-MS data and metabolic networking are all reported in the Materials and Methods. All genomic data has been deposited, as reported above.
